# Different earthquake nucleation conditions revealed by stress drop and b-value mapping in the northern Chilean subduction zone

**DOI:** 10.1038/s41598-024-63015-w

**Published:** 2024-05-28

**Authors:** Jonas Folesky

**Affiliations:** Freie Universität Berlin, Geophysics, Berlin, 12249 Germany

**Keywords:** Solid Earth sciences, Geophysics, Seismology, Tectonics

## Abstract

Stress drop is an earthquake property indicative for the characteristic relation of slip to fault dimension. It is furthermore affected by fault strength, fault topography, the presence of fluids, rupture size, slip, and velocity. In this article, the stress drop image of an entire subduction zone, namely for the seismically highly active northernmost part of Chile, is combined with mapped b-values and their corresponding magnitude distribution in order to better constrain the conditions under which earthquakes of different provenances may nucleate. The underlying recent earthquake catalog contains over 180,000 events, covering 15 years of seismicity, from which more than 50,000 stress drop estimates were computed. Their spatial average segments the subduction zone into different parts, i.e., average stress drop between seismotectonic areas is different, although this difference is small compared to the natural scatter of stress drop values. By considering stress drop variations, b-value map, magnitude distribution, and thermal models, candidate earthquake nucleation mechanisms are identified which can explain the observed distributions. This is done for two exemplary regions: (1) The plate interface, where principally lower stress drop events are found, while at the same time a high spatial heterogeneity of stress drop values is observed. This indicates relatively smooth or lubricated rupture surfaces, and locally it suggests the existence of alternating regions controlled by strong asperities, weaker material, or creep. (2) The highly active intermediate depth (ID) seismicity region, where the variation of stress drop and b-value point to a gradual change of nucleation mechanism from dehydration embrittlement at the top of the ID cloud, over dehydration driven stress transfer in its central part, to thermal runaway shear mechanisms at its bottom. In both cases, the combination of stress drop and b-value distribution helps to better understand the origin and the differences of the observed seismicity.

## Introduction

Earthquake stress drop is basically the ratio of observed slip and characteristic fault dimension^1^. Many applications depend on reliable estimates of source size and stress drop (e.g., for predicting ground motion). It is therefore of high interest to understand the distribution of stress drop in space and time for different geological and tectonic settings. While there is a long debate about the general scale invariance of stress drop, i.e., whether or not the stress drop increases with seismic moment, it is clear that locally the stress drop varies greatly, depending on, for example, the tectonic setting, the availability of fluids, or the roughness of the rupture surface^2–4^. For most medium to small earthquakes it is not easy to obtain reliable stress drop values, as both necessary parameters, the slip and the source size, are not directly accessible. Therefore, the common approach is to derive the source dimension from the corner frequency, by assuming a circular source^5^ and a spectral model^6–8^, fitting this model to the observed and corrected source spectra and then estimating the source size based on the obtained corner frequency^9–11^. Thereby, accurately extracting the source spectrum from which the corner frequency is estimated is a major challenge. Several methods exist to attempt this, using either modeled Greens functions, empirical Greens functions or stacking approaches^9,11–13^. As a result of the broad range of parameter, model and combination options, different stress drop studies are usually hard to compare and results are not univocal^14^. This is why it is important to produce a consistently processed, comprehensive data set which can provide valuable information on the relative differences observed in the target data.

Following this idea, a recently published comprehensive stress drop catalog from northern Chile^15^ is combined with measurements of b-values and the magnitude distribution computed from a recent seismicity catalog for the northern Chilean subduction zone. The catalog covers more than 180,000 events over 15 years of record^16^, which means that it includes the 2007 MW7.7 Tocopilla event and the 2014 MW8.1 Iquique event along with their fore- and aftershock seismicity. The comprehensive stress drop distribution is the first that covers the entire seismically active depth range of a subduction zone (SZ). It consists of 51,510 stress drop estimates and it reveals that the stress drop segments the SZ into different regions, while it also varies strongly on a local scale. In combination with b-value and magnitude distribution, it may allow to better constrain earthquake rupture conditions in different parts of the SZ. In the following, the main features of the stress drop distribution and the here computed b-value map for the northern Chilean SZ are described. Then, two examples of distinct local to regional stress drop observations are selected and specific interpretations on the in situ conditions that may have produced the seismic events are proposed.

## Results

### Stress drop map

**Figure 1:**
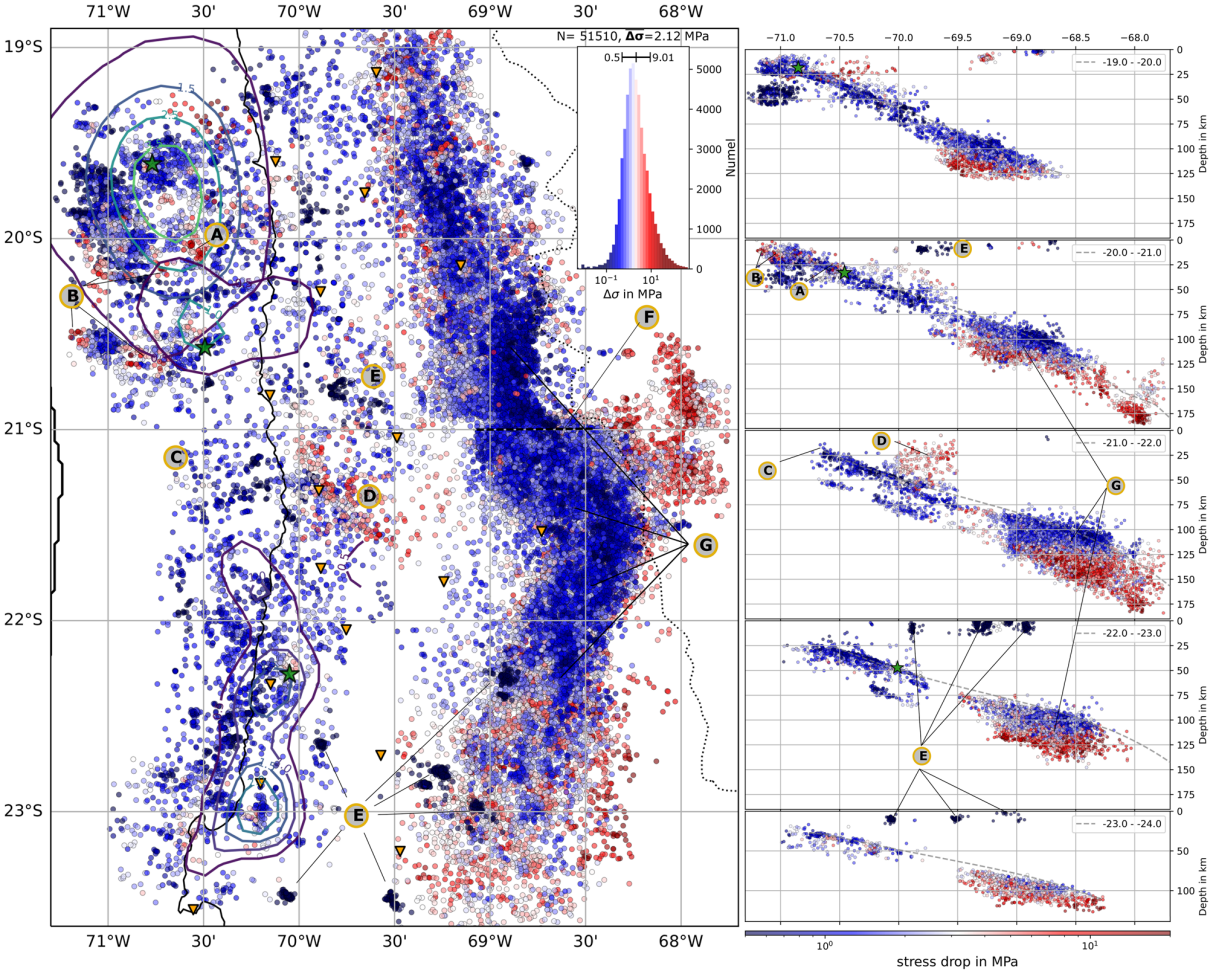
Stress drop distribution map view and E-W depth slices for 1^∘^ latitudinal bins. Coloring is blue to red for low to high stress drop, respectively. The stress drop is smoothed by taking the median over the 10 nearest neighbours. Because of the large amount of seismicity, plotting order is important. In map view, stress drop values are ordered by depth, where shallow events plot on top. Orange triangles depict the IPOC^21^ seismic stations and, green stars, from north to south, are the hypocenters of the M8.1 2014 Iquique event, its biggest M7.6 aftershock and the M7.7 2007 Tocopilla event, together with their slip contours^22,23^. In side view, stress drop plotting order is chronological ascending (see other orders in Figs. S1 & S2). The dashed gray line is the plate interface estimate^24^ averaged for 1^∘^. The average stress drop varies systematically according to seismotectonic region, e.g., between interface events and upper plate crustal events (see Figs. 2 & 3). Smaller scale features are highlighted with capital letters. A: high stress drop patch, coinciding with peak slip region^25^, similarly observed for all nucleation regions (green stars), B: high stress drop clusters coinciding with reported repeating earthquake locations, C: low average stress drop offshore region between the megathrust slip regions and offshore of the Tocopilla event, D: high stress drop continental crust region, E: mining events, F: a gap of the intermediate depth (ID) high stress drop band, separating it into a northern and southern part. Much clearer visible when high stress drop events are plotted on top (see Fig. S1). G: highly active ID seismicity cloud of increasing median stress drop in the direction perpendicular to the interface. The map was created using Matplotlib^26^ v3.5.1 & Cartopy^27^ v0.20.2.

**Figure 2:**
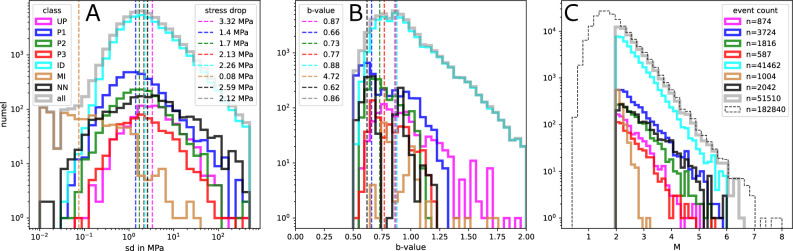
Histograms for stress drop, b-value and magnitude distributions, broken down by event class. Coloring corresponds to the inset in Fig. 3A. Grey is the sum of all events. (**A**) Stress drop histogram and class wise median stress drop; (**B**) b-values for each class; (**C**) magnitude distribution, here, the thin black dashed line is the complete seismicity catalog^16^. Note, that stress drop and b-values for the MI class events are highly artificial, because of their non-natural source type and magnitude distribution.

**Figure 3:**
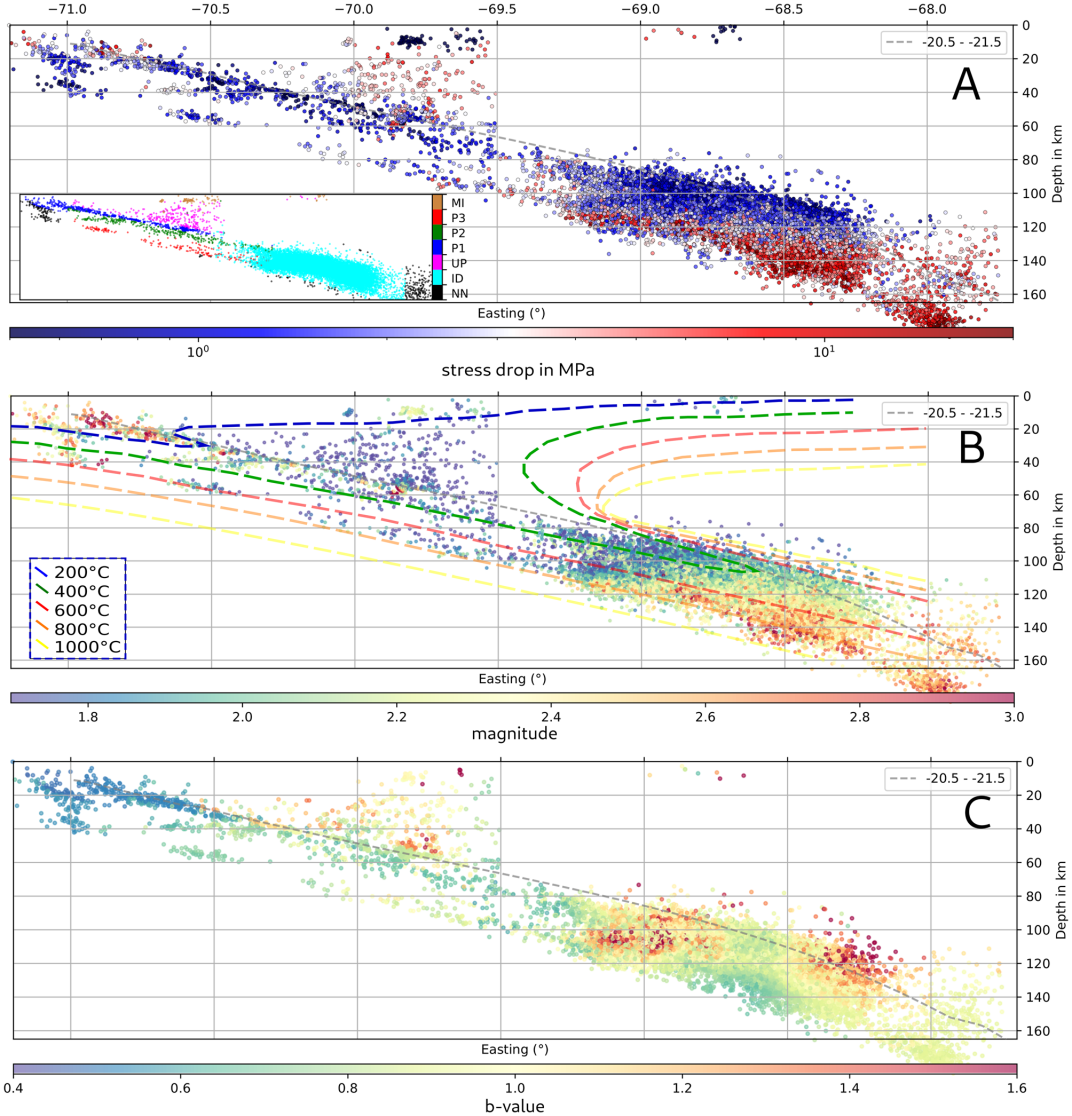
Comparison of stress drop, magnitude and b-value distribution. (**A**) Stress drop distribution side view for events between − 20.5^∘^ to − 21.5^∘^. The inset shows the event classification of events based on occurrence region^28^. (**B**) magnitude distribution side view for events between − 20.5^∘^ to − 21.5^∘^. Overlain is a thermal model of the SZ^29^. (**C**) b-value distribution for the same data. The dashed gray line in all sections is the local plate interface^24^. A and B are smoothed over the 10 nearest neighbours. Note, that, because of the large amount of events, plotting order is highly important. Here, events are plotted in chronological order (see Figs. S1 & S2 for others).

Database for this work is the recent stress drop map from Folesky et al. 2024^15^ (Fig. 1), who employed the spectral decomposition method^11,13,17^ and the spectral ratio method^9,12,18^ to compute a comprehensive set of 51,510 stress drop estimates for northern Chile. The data set displays that, principally, the stress drop variability is high. It shows a log-normal distribution for all estimates, as well as for each spatial subset of events (Fig. 2A). 97.5$\%$ of the estimates fall in the range between 0.1–100 MPa. The stress drops in this data set scale with moment ($\sim M_0^{0.5}$) similar to other studies using comparable approaches^17,19,20^ (see supplement Fig. S3) but in contrast to others^2,11^. Scale dependence is, at least partially, a result of the processing parameters^20^, and thus, an additional stress drop map with the average scale dependency removed is given in Fig. S4, which displays that the overall spatial variability remains robust but relative differences decrease. A direct comparison to the magnitude distribution reveals both, areas of highly similar variation and strong deviations between magnitude and stress drop map (Fig. 3).

Following the event classification of the seismicity catalog^16^, the events are divided into the location based classes: upper plate (UP), interface (P1), upper plane (P2), lower plane (P3), intermediate depth (ID), mining (MI) and else (NN) as depicted in Fig. 3A. The statistics of each class are summarized in Fig. 2A–C. The class wise stress drop median values vary between 1.4 MPa (P1) and 3.32 MPa (UP) for natural seismicity while MI class events have an estimated median stress drop of only 0.08 MPa. Figure 3A displays a depth section of the subduction zone, where the segmentation of stress drop is apparent. Lowest values are found for the surface mines (MI) and the interface class events (P1) but also for the top layer of the ID event cloud at locations close to the estimated interface extension at depth^24^. Events at greater distances to the interface show increased stress drop (P2, P3 and especially UP class). The events at the bottom of the ID class appear to have the highest average stress drop, extending from about 115 km to about 180 km depth in a band parallel to the plate interface. The distribution of stress drop within each class shows local variability, as shown in Fig. 1. Because of the large amount of data points, the plotting order is of high importance. Here, the shallowest events are plotted on top (Two other versions with high stress drop and low stress drop on top are displayed in the supplement Figs. S1 & S2). There are several smaller scale stress drop variations such as locally elevated stress drop at nucleation and high slip regions of major earthquakes (green stars & (A) in Fig. 1), confined to known repeating earthquake locations (B), or a limited crustal region (D) of strongly increases seismic activity^24^. Regions of comparatively low median stress drops are found at the shallowest interface, in the offshore region west of the Tocopilla event, and in the unbroken gap between the megathrust events (C). The intermediate depth seismicity cloud (G) consistently shows low stress drop on its top, with increasing values further from the interface towards its bottom. In the northern part, a high stress drop band is found between 69^∘^–69.7^∘^ at 100-120 km depth (cf. Fig. S1). A clear gap (F) separates this band from its southern part, where high stress drop values are found 20 km further east and extending to much greater depth. Please note that despite all efforts to improve robustness, stress drop values may be biased by inaccuaries in the velocity model, change of the shear wave velocity to rupture velocity ratio or changing rupture dircetivity^15,30^.

### b-value distribution

**Figure 4:**
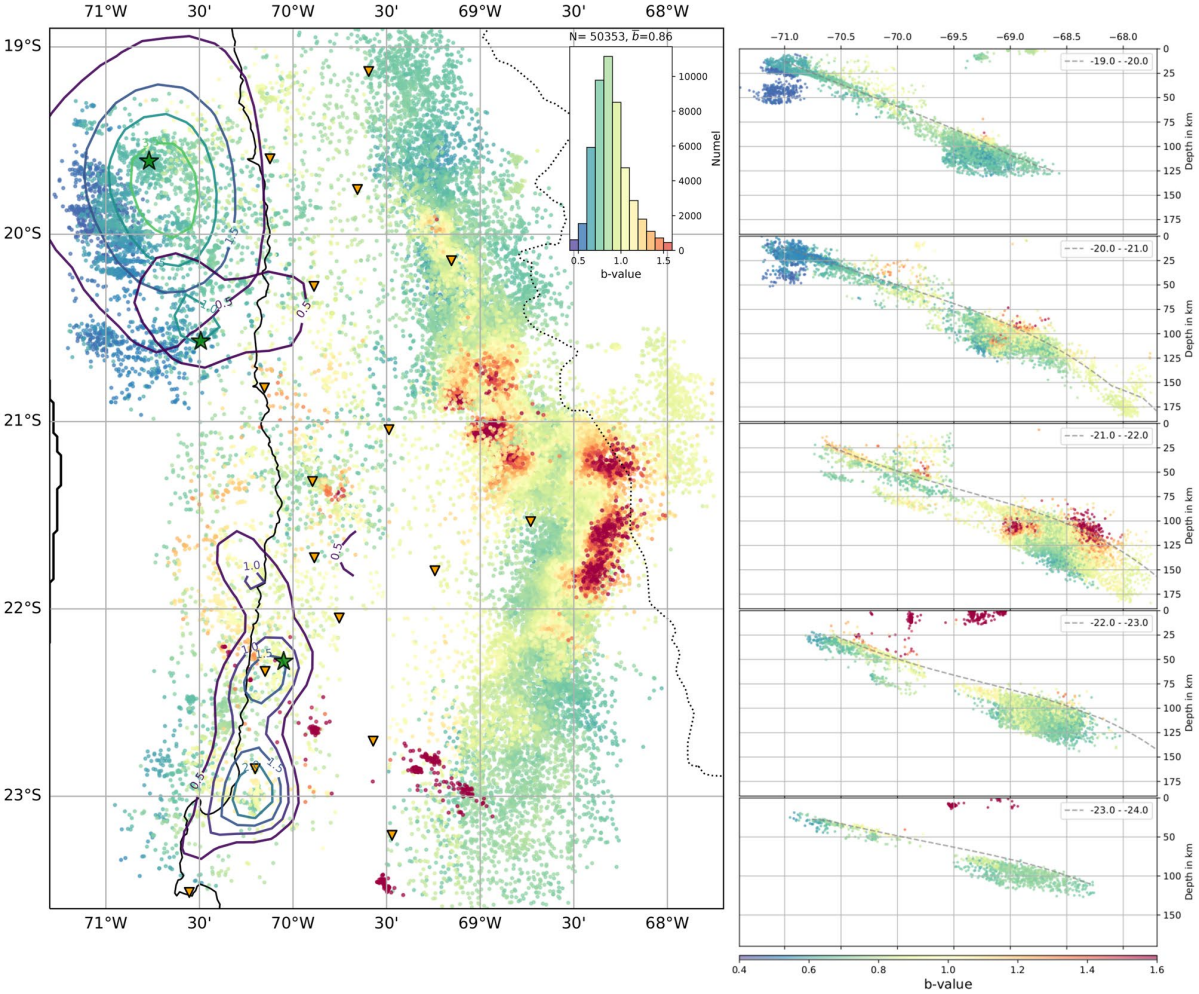
b-value distribution in map view and E-W depth slices for 1^∘^ latitudinal bins. b-values are computed for each event and its minimum 200 nearest neighbours, and the value is directly assigned to the event location. Coloring is blue to red for low to high b-value, respectively. Plotting order in map view is based on depth, with shallow events plotting on top. The side view show events in chronological order. No additional smoothing is applied. Highest b-values are found at mining locations (see Fig. 1) and on top of the ID cloud at 100±20 km depth. Low b-values are seen at the bottom of the ID cloud and along the shallow plate interface, upper and lower seismicity planes at shallow depths. The slip regions of the megathrust earthquakes show spatially varying b-values, with elevated estimates predominately located close to high slip regions. Uncertainties for each data point are given in Fig. S5. This map was created using Matplotlib^26^v3.5.1 & Cartopy^27^v0.20.2.

b-values were mapped by applying the maximum-likelihood method (MLE)^31^ using 200-1000 of the nearest neighbours of each event, limited to a 50 km radius, i.e., for each event an individually computed b-value is obtained. The map is shown in Fig. 4. The associated standard deviations, obtained from bootstrapping, are displayed in the supplement (see Methods, Fig. S5). The b-values show large variability throughout the SZ. The median values for the classes are separated by a few percent and range from 0.66(P1) to 0.88(ID), with the MI class events sticking out with an average b-value of 4.72 (see Fig. 2). Note, that these values compare well to the classwise and independently obtained estimates for the entire dataset^32^. Inside each class, variability is higher than the differences between classes. The map in Fig. 4 clearly reveals mining event locations in dark red, corresponding to high b-values ($b\ge$1.5). The shallow interface, especially in the Iquique event rupture region, is dominated by principally low b-values. Also, the most shallow offshore region south-west of the Tocopilla megathrust event appears to be low b-value. In the ID band, one finds high b-values on top of the ID cloud, mostly limited to a depth of 80-100 km in the part north of 21^∘^S. To the south, high b-values are shifted to the east and reaching to greater depths, always on top of the high density seismic cloud. In contrast to that, the bottom of the ID cloud shows a significantly decreased b-value.

## Discussion

In the results section, several observations, ranging from localized stress drop variability to larger scale variations, seen throughout the subduction zone, were listed. In the following, stress drop and b-value information will be combined to identify characteristics of earthquake nucleation in selected areas of the SZ. Two examples are picked for further discussion: (1) the interface (P1) is dominated by predominantly low stress drop events while its stress drop distribution simultaneously is heterogeneous and (2) the principle variation of stress drop inside the intermediate depth seismicity (ID) cloud.

(1) The interface seismicity is dominated by low stress drop values. From Figs. 1, 2A & 3A one may note that the interface has the lowest median stress drop compared to all other event classes (except mining). In the depth sections in Figs. 1 and 3A it appears as a band of blue dots, in large parts clearly separated from crustal events (UP) and also from upper plane events (P2). This is in line with observations that subduction megathrusts must be weak faults^33^ because of their unfavorable orientation for failure. A plausible explanation for the low stress drop might be the high water content at the interface and in the entire crust of the subducting plate^34^, which also should affect the upper plane seismicity, and which effectively lubricates the potential rupture surfaces either by directly increasing pore pressure and reducing effective differential stress or by facilitating the genesis of partial melt during sliding more easily. Another possible reason for low stress drop is the maturity of the rupture surfaces and thus their topographic properties^35^. Especially at the plate interface, the decreased roughness of the event rupture surfaces from repeated slip should result in comparatively low stress drop ruptures^4^. There is no pronounced depth dependence for interface event median stress drop^15^. Also, one should note that in principle at the plate interface a substantial variability of stress drop is observed, i.e., there exist low and high stress drop regions, indicative for the large heterogeneity of the interface rupture conditions (Fig. 1). Note furthermore, that despite the fact that interface seismicity ceases below 65 km depth, a large group of events with similar stress drop level occurs at the top of the ID seismicity cloud and extents down to about 120 km (e.g., Fig. 3A).

The interface stress drop distribution is heterogeneous. For example, one finds localized high stress drop areas at multiple locations on the plate interface. Several of them are marked (B) in Fig. 1. They coincide with known locations of repeating earthquake (RE, repeaters) series^36,37^. These areas were described as parts of west-east extending lineaments of seismic events called streaks^37^. While repeaters located on the high slip patches of large events have been shown to exhibit low stress drop^38^ other studies report relatively high stress drops for long-lasting repeater sequences^30,39^. Indeed, in northern Chile the repeater sequences localized at high stress drop areas are located adjacent to, not on the high slip patches, and they were already active before the Iquique event^36^. Possibly, the high stress drop estimates for those REs are an expression of the elevated slip to fault dimension. The asperity can only host limited size events. To actually match the macro-scale plate convergence rate, either high slip, multiple slip events, large amounts of aseismic slip on the asperity are needed, or a combination of them^40,41^. The b-value estimates for several of those asperities are very low, with b < 0.8. Low b-values at asperities might be explained by a high level of differential stress^42,43^ or by the events’ spatial limitation (lower fractal dimension of the events) which also has been associated with low b-values^44^. Also note, that b-values may be strongly influenced by the 2014 Iquique event fore and aftershock series, which was not considered, here.

South of the asperities between 20.8^∘^S and 21.4^∘^S GPS studies describe the interface as potentially locked^22^ but this has been questioned based on bathymetry and gravitational estimates^45^. Other authors report a much lower degree of locking^46^ and even creep was suggested^47^. Indeed, directly south of the neighbouring seismically more quiet gap around 21^∘^S, estimated b-values are higher (but few), which might support a creeping section^48^. Corroborating this, the (few) stress drop estimates from this region are relatively small, which is indicative for potentially lubricated or smooth rupture surfaces^4^. Additionally, no single patch of locally elevated stress drop is found in this area.

Hence, by combining the geophysical hints obtained from different techniques, a creeping section between 20.8^∘^S and 21.4^∘^S also appears to be a plausible scenario. Note, that albeit almost all available locking studies report a high degree of locking south of 21.4^∘^S, stress drops and b-values do not show abrupt changes, here. Also note, that this work does not consider temporal variability of stress drop inflicted by the megathrust events, as they pose an additional subject and will be studied in a separate paper. According to Folesky et al. 2024^15^, there does not appear to be a long term offset in stress drop induced by the events but only a short (few days) increase of stress drop after a big event, localized to the direct rupture vicinity. The intermediate depth seismicity appears not affected.

(2) In northern Chile the double seismic zone blends into a 20–30 km thick cloud of ID seismicity below 80 km depth, filling up the gap between upper band and lower band of the Wadati-Benioff-Zone(WBZ)^28^(Fig. 1). The ID cloud continues to depths of 220 km. It comprises about 80$\%$ of the entire seismicity from the catalog, and relatively higher median stress drop is observed, here. Again, stress drop is distributed heterogeneously, but a consistent increase with distance to the interface is observed (Fig. 3). At the top of the ID cloud stress drops are low ($\sim$1 MPa), while the events in the center show medium stress drops (2–4 MPa), and high stress drop (20 MPa and more) is localized all along the lower bound of the cloud in the oceanic mantle. Simultaneously, median stress drop increases from about 2 MPa at 100 km depth to 15 MPa at 180 km depth^15^.

The b-value also shows significant changes throughout the ID cloud. At the top of the ID cloud, the b-value is 1.2 and higher, which are values often reported for earthquake swarms associated with magma chambers beneath active volcanoes^49,50^. Hence, it is possible that at the ID top layer fluid from below accumulates, increases pore pressure and induces melting in the overlying mantle. Localized high b-values in a similar depth range and on the top of the WBZ were also reported in other SZs^51,52^ where they were interpreted as results from pore pressure increase due to dehydration processes. In contrast, at the bottom of the ID cloud, low to very low b-values are found, possibly indicating a more dry environment, dominated by high differential stresses induced by slab densification and increased slab pull^32^.

Hence, one interpretation of the b-value map in Figs. 3 and 4 is that the available water content is likely to change from top to bottom, from abundant to more dry. This would be in line with a highly hydrated oceanic crust (high degree of serpentinization) and a less hydrated oceanic mantle where hydration may stem from deep spreading faults at the mid-ocean ridge or subduction related bending faults near the trench, which would also explain the decay with depth^53^. Simultaneously, stress drops increase from low to high from top to bottom, much like mean magnitude.

These combined observation might be an expression of a gradual change of the predominant nucleation mechanism. There are several candidate mechanisms that have been proposed to be seismogenic at ID seismicity depth: dehydration embrittlement^54^, where brittle yielding of the rocks due to fluid overpressure from dehydration of mineral phases would cause earthquakes; dehydration driven stress transfer^53^, where mechanical instabilities could nucleate due to high stress intensity at the tip of localized antigorite clusters in olivine-antigorite aggregates and thus any unstable minor phase could locally induce a critical stress transfer leading to an earthquake; and thermal shear instability^55^, where highly localized viscous creep leads to an increase in temperature, weakening, and consequentially to self-sustaining further slip in a narrow shear zone.

For example, in the Alaska SZ dehydration embrittlement has been proposed as a possible mechanism for the shallow, higher b-value part of the ID cloud^51^, similar to Hokkaido, Japan, where the shallower part of the ID cloud, the oceanic crust, showed higher b-values and the deeper parts of the ID cloud, the oceanic mantle, showed lower values which was interpreted as signs of dehydration embrittlement and dehydration driven stress transfer as dominating rupture mechanisms, respectively^56^. The authors pointed out that the large variety of phase transitions which allow dehydration driven stress transfer also cover a relatively broad P-T window of 550–700^∘^C. In Chile, those values are present in the central part of the ID cloud^29,57^ (Fig. 3B). For a deep-seated ID nest in Colombia, thermal runaway shear instability was identified as a likely mechanism^58^. Their local depth is 140–160 km with temperatures between 600–1000^∘^C and stress drops between 10-100 MPa, which are conditions similar to those from the deepest parts of the ID cloud in northern Chile. To obtain sufficient delta T, a suitable combination of layer thickness (fault width) and slip is needed, and found possible for events of M>=4, especially for dry rock conditions. Smaller events cannot reach the necessary delta T, because not enough frictional heat can be released. In our data set, the high stress drop, the low b-values, and the increased mean magnitude localized at the bottom of the ID cloud would allow a similar hypothesis. Thermal models for the northern Chile subduction zone report temperatures isotherms of 600–800^∘^C for the lower plane seismicity reaching up to 1000^∘^C at the lower bound of the ID cloud (see Fig. 3). If this is indeed the predominant nucleation mechanism at the ID cloud bottom, one would expect that with increasing hydration in the oceanic mantle in direction towards the plate interface, thermal runaway gets less likely^58^ and the other processes might dominate. This could then explain both, the decreasing mean magnitude and the decreasing median stress drop, observed when crossing the ID band from bottom to top. In its central part dehydration driven stress transfer could be dominating, which is possible over a variety of P-T combinations and does not necessarily require high amount of fluids^59^ while at the top of the ID cloud dehydration embrittlement appears to dominate as indicated by the high b-values and low stress drops.

## Conclusions

This work combines stress drops, b-values, and magnitude variability to observe and discuss the heterogeneity of rupture nucleation conditions in the northern Chilean subduction zone, a region which is characterized by high seismic activity. The rich data allows studying rupture nucleation conditions from the shallow interface down to intermediate depths of over 200 km. By combining information from a recent stress drop study with a newly composed b-value map, the magnitude distribution and an available thermal model, it becomes feasible to explain local variability of earthquake nucleation mechanisms as well as general differences between earthquakes of different classes. This is done for two particular examples.

First, for events of the plate interface seismicity class, principally low stress drops are observed. This is in line with the weak fault hypothesis, and it is interpreted as a signature of a relatively smooth or highly lubricated rupture surface. At the same time, potential asperities and a creeping region at the shallow interface, indicated by locally strong variations in stress drop, are found. This resembles the strong heterogeneity of the shallow interface.

The second area of investigation is the highly active intermediate depth seismicity band, where an interface-distance dependent stress drop increase is found supported by the variation of b-value in the area, indicating a dry environment at the ID cloud bottom and an abundance of fluid at its top with gradual changes in between. This observation is interpreted as an expression of a possible change of the dominating nucleation process from dehydration embrittlement at the uppermost parts of the ID cloud, to dehydration driven stress transfer at its central part and to self localizing shear heating at the foot of the ID event cloud.

For both exemplary cases, the combined analysis helps to better constrain the localized earthquake nucleation properties, and thus it adds to an improved understanding of the subduction zone processes in general. Again, it is noticeable that the large and consistently processed data sets of magnitudes, b-value and stress drop distribution used in this work is the sound base that allow interpretation of such kind while the direct comparison of results from different studies remain problematic due to the many parameter choice and techniques available.

## Methods

### Stress drop

This work is based on the comprehensive stress drop map from Folesky et al. 2024^15^, who employed the spectral decomposition method^11,13,17^ to calculate corner frequencies. For comparison, they also applied the spectral ratio method for suitable events^9,12,18^. Stress drops were then calculated using the circular fault model^5^ where the radius was replaced by $r =\frac{f_c}{k*\beta }$^6^:

1 $$\begin{aligned} \Delta \sigma =\frac{7\pi \mu \overline{D}}{16r}=\frac{7M_0}{16r^3}=\frac{7}{16}\left( \frac{f_c}{k\beta }\right) ^3M_0. \end{aligned}$$

Here $\mu$ is rigidity, D is average slip, $M_0$ is seismic moment, $f_c$ is corner frequency, the constant k relates to the spherical average of the corner frequency for a specific theoretical source model, and $\beta$ is the shear wave velocity at the source used as a proxy for rupture velocity. Details on band limits and error estimation can be found in Folesky et al. 2024^15^. Note, that in this work, all figures displaying spatial stress drop distributions are smoothed by computing the median over 10 spatial nearest neighbours, which significantly enhances visual perception of stress drop variability.

### b-value

For b-value computation, the maximum-likelihood method (MLE)^31^ as implemented by Goebel et al. 2017^44^ was used. b-values were computed based on a selection of the 200-1000 nearest neighbour events in the respective class, depending on availability, within a maximum spatial distance of 50 km. For better consistency, a fixed value of $M_c$=2.69^32^ was used for the processing. The obtained b-value is then associated to the location of the initial event. The procedure is repeated for all events in the original earthquake catalog^16^. Events with insufficient number of neighbours obtain no b-value. In a second step, and to obtain the best comparability to the stress drop map, the results are reduced to those events where stress drop estimates were reported. The standard deviation for each b-value is obtained from bootstrapping. For this, the MLE b-value estimate is recomputed for 100 realizations of the resampled minimum 200 nearest neighbours, where 10$\%$ of the events are randomly neglected. It is summarized in map and histogram in the supplement (Fig. S5) as well as in the separate b-value catalog file (see Data Availability). Note, that the median of the b-value distribution (Fig. 2) compare well to the directly classwise obtained estimates^32^. Also, the selection radius can be considered as a smoothing kernel, which limits the spatial resolution of the b-value map.

### Magnitudes

From the seismic catalog^16^ the MA calibrated magnitudes^60^ were used which have been tested to compare very good to the scalar seismic moment^15^, hence, they are treated as moment magnitudes. To obtain a spatially better perceptible image, for display, values were smoothed using the mean of the MAs of the 10 nearest neighbours of each event. The obtained value is then assigned to the original event location. Similar to the b-value computation, the complete seismicity catalog for computing the mean magnitude was used, but results were limited to those events present in the stress drop catalog.

### Supplementary Information

Supplementary Figures.

## Data Availability

The northern Chile seismic catalog^16^ is available at https://doi.org/10.5880/GFZ.4.1.2023.004; the stress drop catalog^15^ is available at https://doi.org/10.5281/zenodo.10400960, the b-value catalog is available at https://doi.org/10.5281/zenodo.11208068.
