# Supplementary material for: Different earthquake nucleation conditions revealed by stress drop and b-value mapping in the northern Chilean subduction zone
Source: Sci Rep. 2024 May 28;14:12182. doi: 10.1038/s41598-024-63015-w (PMC11133448; doi:10.1038/s41598-024-63015-w)
Supplement: Supplementary file 1 — Supplementary Figures. [file 41598_2024_63015_MOESM1_ESM.pdf]

Supplement of
Different earthquake nucleation conditions revealed by stress drop and b-value mapping in the northern Chilean subduction zone

by Jonas Folesky,
Freie Universität Berlin, Geophysics, Berlin, 12249, Germany,
jonas.folesky@geophysik.fu-berlin.de

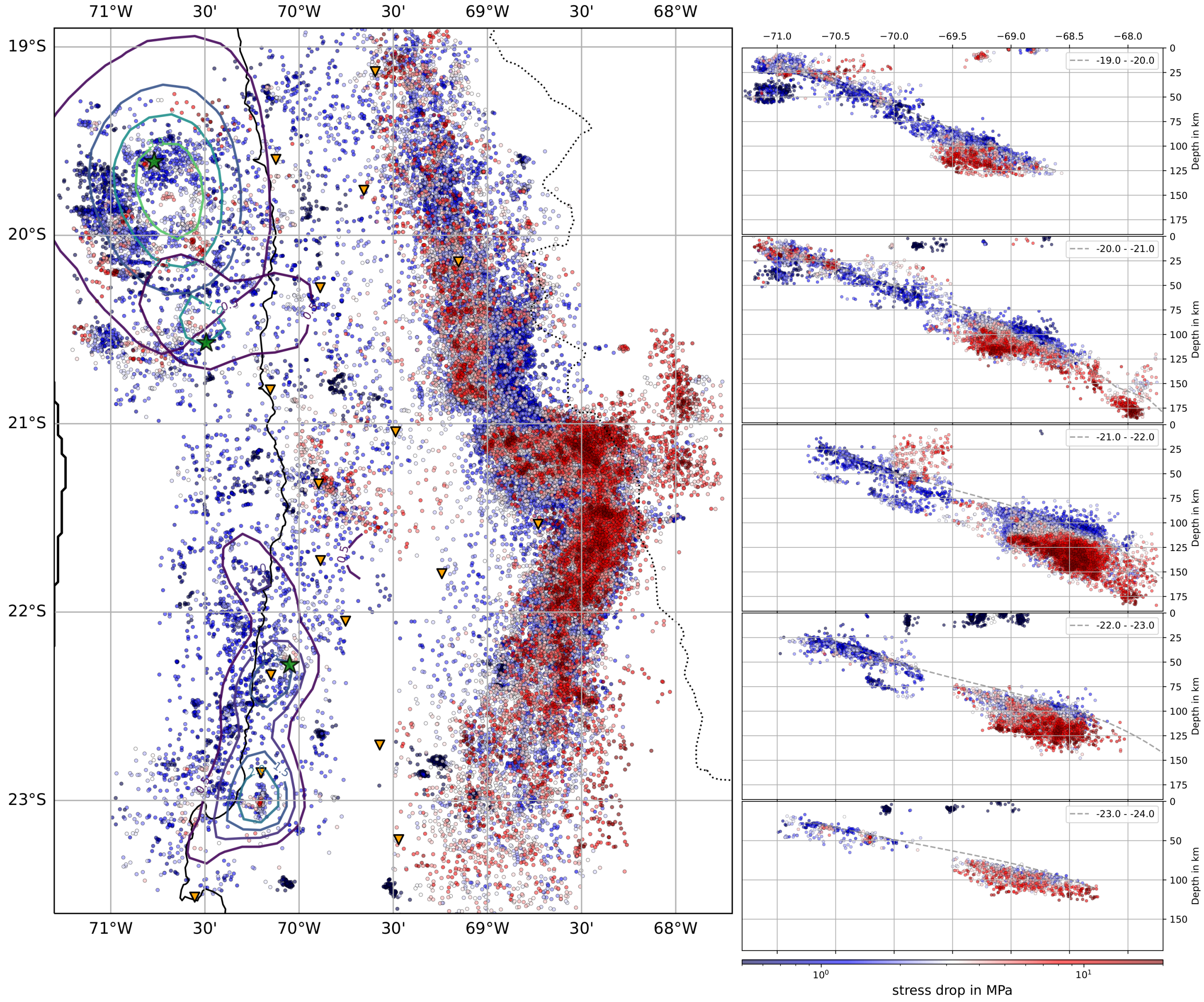

Figure S 1. Stress drop distribution map view and E-W depth slices for 1° latitudinal bins similar to main manuscript Fig.1. but plotting order of points is varied. Here, the **highest stress drop** events plot on top for both, map and side views. This map was created using Matplotlib v3.5.1 & Cartopy v0.20.2.

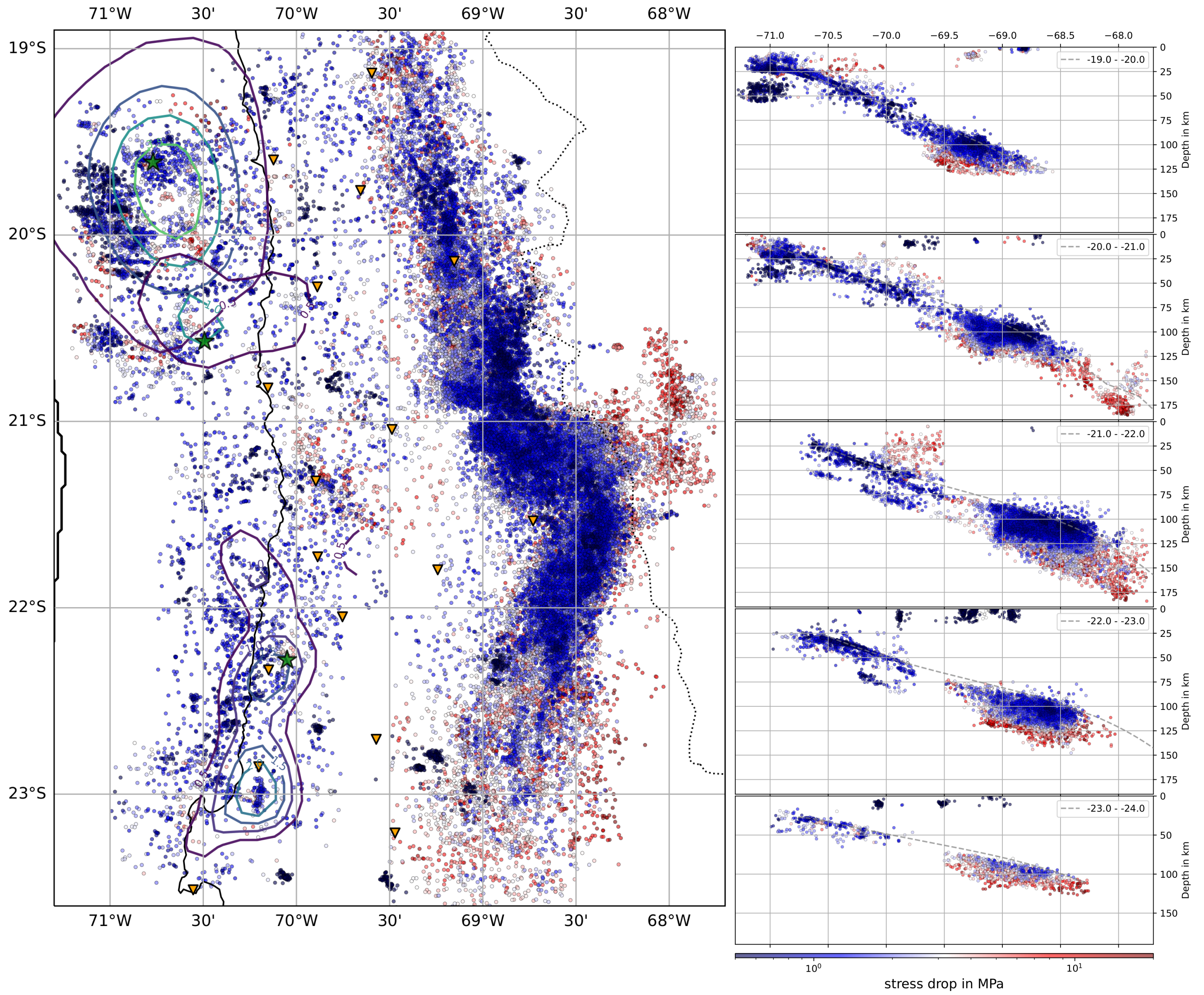

Figure S 2. Stress drop distribution map view and E-W depth slices for 1° latitudinal bins similar to main manuscript Fig.1. but plotting order of points is varied. Here, the **lowest stress drop** events plot on top for both, map and side views. This map was created using Matplotlib v3.5.1 & Cartopy v0.20.2.

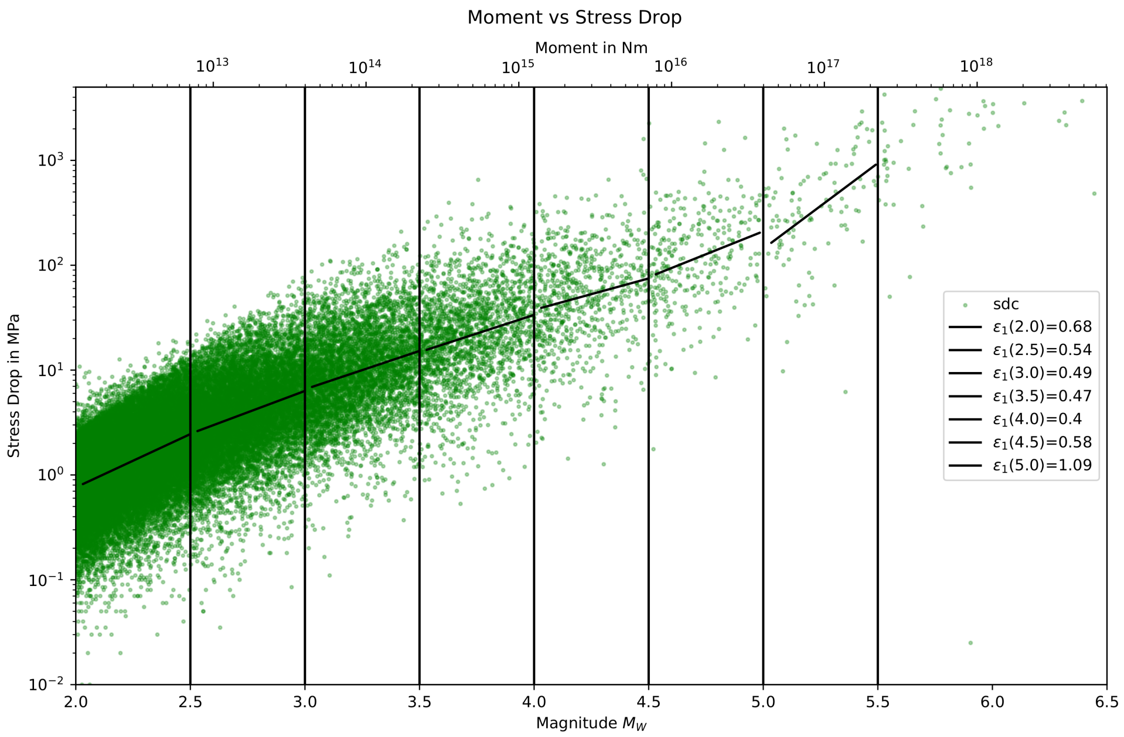

Figure S 3. Stress drop scaling with seismic moment in the data is about $\Delta\sigma \sim M_0^{0.5}$. The scaling is a result of the applied stacking technique. One can correct for the average scaling. The corresponding map is shown in Fig.S4.

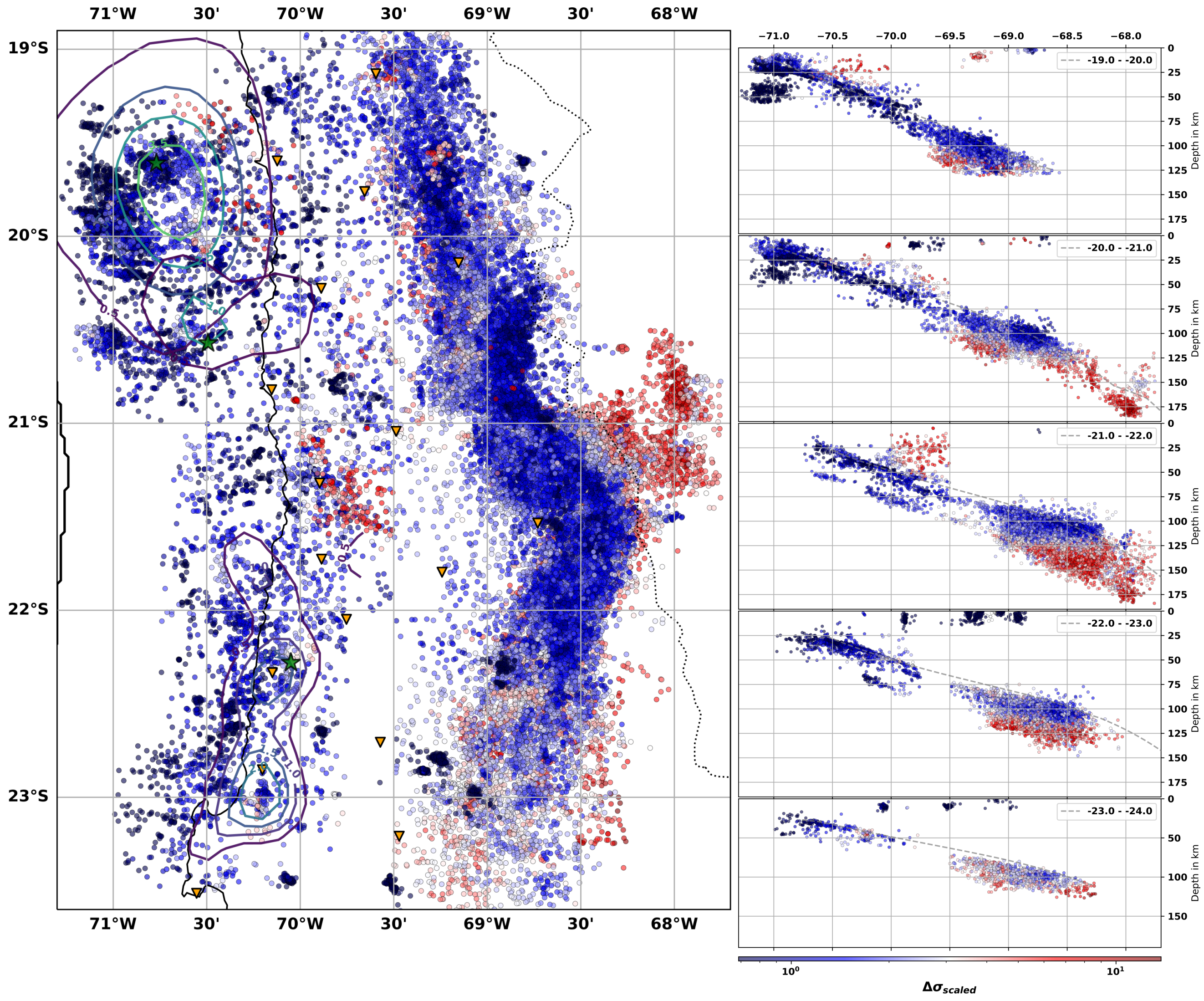

Figure S 4. Stress drop scaled with moment map view and E-W depth slices for 1° latitudinal bins similar to main manuscript Fig.1. Each individual stress drop value was corrected by the moment dependence as $\Delta\sigma_{scaled} = \frac{\Delta\sigma}{M_0^{0.5}} * \frac{\overline{\Delta\sigma}}{\overline{(\Delta\sigma/M_0^{0.5})}}$, where the overline resembles the median of the given property. The result is highly similar to Fig.1 from the main manuscript. Plotting order is the same as in Fig.1. This map was created using Matplotlib v3.5.1 & Cartopy v0.20.2.

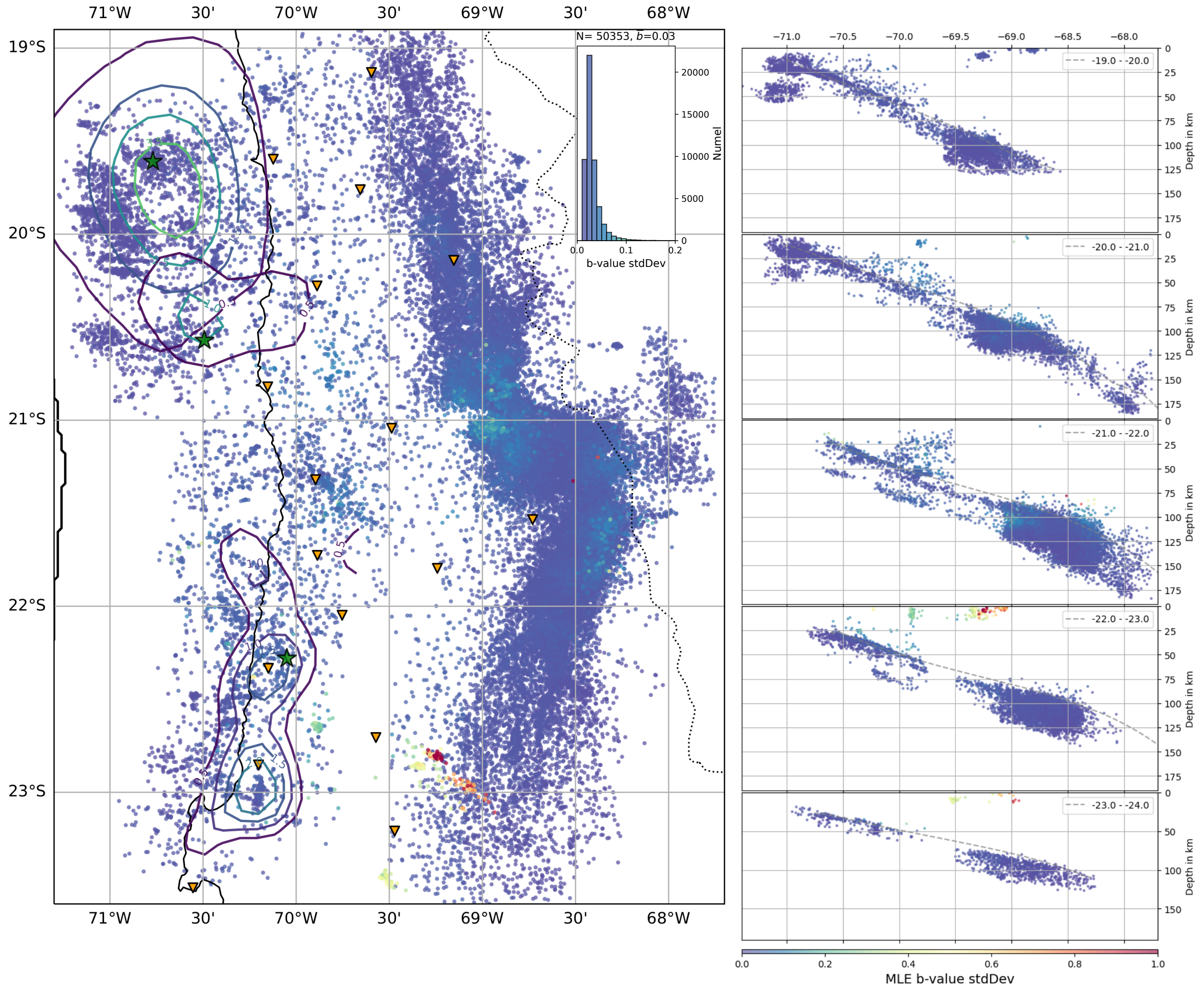

Figure S 5. b-value standard deviation corresponding to Fig.4 main manuscript. As b-value is computed for each point in Fig.1 separately, each point also has a corresponding b-value STD, computed from 100 bootstrapping runs for each. This map was created using Matplotlib v3.5.1 & Cartopy v0.20.2.
